# Smoking Status as a Predictor of Hip Fracture Risk in Postmenopausal Women of Northwest Texas

**Published:** 2007-12-15

**Authors:** Anne V Denison, Marjorie R Jenkins

**Affiliations:** Texas Tech University Health Sciences Center, Clinical Research Unit. Ms Denison is also affiliated with the Amarillo Veterans Administration Health Care System, Amarillo, Texas; Women’s Health Division, Texas Tech University Health Sciences Center, Amarillo, Texas

## Abstract

**Introduction:**

The purpose of this study was to determine the effect of cigarette smoking on the risk of hip fracture for postmenopausal women living in rural and urban areas of Northwest Texas.

**Methods:**

Using an unmatched case-control design, we compared postmenopausal women who had recently experienced osteoporotic hip fracture with women who had not. Both study groups completed a questionnaire on demographic, clinical, and behavioral risk factors for osteoporotic hip fracture. We categorized smoking status as *never smoked*, *former smoker*, and *current smoker*. Covariates included age, weight, age at menopause, physical activity, estrogen replacement, calcium supplementation, and rurality. We used univariate and multivariate logistic regressions to test the associations between hip fracture and the independent variables of interest.

**Results:**

We found an increased risk of hip fracture for former smokers (adjusted odds ratio [OR], 2.27; 95% confidence interval [CI], 1.22–4.21) and current smokers (adjusted OR, 3.72; 95% CI, 1.59–8.70). Residence in a rural county (population <100,000) also was associated with increased risk (adjusted OR, 2.71; 95% CI, 1.48–4.95).

**Conclusion:**

Former and current smoking increased the risk of hip fracture in this population of postmenopausal women.

## Introduction

Hip fracture due to osteoporosis is a major cause of morbidity and mortality among postmenopausal elderly women. The incidence of this type of hip fracture is rising, in part because this risk group continues to grow: the U.S. population aged 65 to 84 years is predicted to increase by 114% during the next 50 years, and the cohort aged 85 or older, by almost 400% (1). Along with the aging population and the increasing incidence of osteoporosis comes rising cost. Rates of hospitalization for hip fracture are increasing despite advances in research, increased resources, and improved diagnosis and treatment of osteoporosis. Annual medical costs related to osteoporosis are expected to approach $200 billion by 2040 (2).

To focus both clinical and public health prevention efforts most effectively, a thorough understanding is needed of the risk factors for hip fracture. Many studies show that low bone mineral density (BMD) is not the sole predictor of fracture risk (3). We have little control over major risks for osteoporotic hip fracture such as age, comorbid conditions, and genetic factors. Other risk factors, including smoking, diet, and physical activity, however, are potentially modifiable at the population level. Although clinical intervention based on BMD testing may significantly reduce risk at the individual level, population-based interventions are needed to target modifiable health behaviors.

A number of studies have identified a modest association between cigarette smoking and the risk of hip fracture (4-7). Although scientists hypothesize that part of this effect may result from decreased osteoclast activity, reduction in BMD explains only about 23% of the smoking-related risk for hip fracture (8). Furthermore, the complex relation between smoking and BMD is potentially confounded by other factors, including physical activity, weight, diet, and age at menopause. The purpose of this study was to clarify the role of cigarette smoking as a predictor of risk for hip fracture in a population of postmenopausal women in Northwest Texas.

## Methods

### Study design and setting

We used an unmatched case-control study design to test the association between smoking and osteoporotic hip fracture. To control for potential confounders, we obtained demographic, geographic, clinical, and health behavior characteristics from respondents. We recruited case patients from two large general hospitals, both of which provide emergency care, orthopedic surgery, and rehabilitation options, services that make them good sites for identifying patients with hip fracture. These hospitals are part of a regional medical center serving metropolitan Amarillo and a wide area of rural counties in Northwest Texas. Most patients from the region who experience hip fracture are referred to one of these two hospitals.

Patients admitted with a diagnosis of fracture of the head or neck of the femur were eligible for inclusion in the study if they were female, 50 years of age or older, mentally alert, and not residing in a nursing home before their fracture. Women with a previous history of hip fracture and women with hip fractures of pathologic etiology, such as Paget disease of bone and metastatic bone cancer of the femur, were excluded from participation. For the control group, we selected a convenience sample of women visiting their primary care practitioners in the internal medicine clinic of an academic medical center. We applied the same inclusion and exclusion criteria to the control group as we did to the case group. We enrolled approximately 1.5 controls for each case patient. Participation in the study was voluntary.

The Institutional Review Board of the Texas Tech University Health Sciences Center, Amarillo, approved this study. The study was exempt from requiring informed consent because risk to participants was minimal and data were purged of personal identifiers.

### Study procedures

Using a 68-item questionnaire, we collected data from both study groups from July 2004 through April 2006. A registered nurse served as study coordinator, and orthopedic and rehabilitation floor staff identified potential case patients. The study coordinator visited the two hospital sites daily to interview potential case patients and assess their mental status, willingness to participate in the study, and eligibility for inclusion. Questionnaires were completed by either the study coordinator or the patient, with assistance from family members as needed. To maximize the response rate and ensure that patients were as lucid and pain-free as possible when interviewed, the coordinator visited potential case patients up to four times during their hospital stay. Control patients received a copy of the survey from the receptionist as they signed in to see their care provider and placed completed surveys in a covered box in the reception area. All surveys were anonymous. We created a database of the questionnaire responses in Microsoft Office Access 2000 (Microsoft Corporation, Redmond, Washington).

We identified 418 potential case patients at the two hospitals, and of these, 297 (71%) met the inclusion criteria. The remaining 29% were ineligible because of mental status, residence in a nursing home, or the nature of the need for treatment. Conditions rendering a patient ineligible primarily comprised fractures of the lower femur and nonemergent hip replacements. Of the eligible patients, 65% completed questionnaires, approximately 11% refused to participate, and 24% were lost to follow up because the patient was discharged before completing the survey form, was transferred to a higher acuity setting (i.e., intensive or critical care units), or died. We excluded the surveys of three patients residing outside of the geographic boundaries established for the study. A total of 190 case patients were included in the study. We distributed approximately 350 surveys to potential control patients and received 309 completed surveys (88% return rate). Of these questionnaires, 298 contained data on smoking status and were included in our analysis.

### Measures

The primary dependent variable was hip fracture. We used this dichotomous variable for outcome in both the univariate analysis and the multiple logistic regression models. We used current smoking as a secondary outcome variable to test differences in the distribution of characteristics on the basis of self-reported smoking status.

The independent variable of interest was self-reported smoking status, as determined by answers to two survey questions: Did you ever smoke? Do you smoke now? We entered responses into three categories: *never smoked*, *former smoker*, and *current smoker*. Controlling variables, based on review of the literature, were age in years (50–64, 65–79, ≥80), weight in pounds (<127, ≥127), exercise patterns (≤2 times/week, >2 times/week), age in years at menopause (≤34, 35–44, 45–51, ≥52), history of hormone replacement therapy (ever, never), calcium supplementation (yes, no), and alcohol consumption (yes, no).

The final variable of interest, rurality, or the degree to which a living environment has a rural quality or character, was based on the population size of the county in which a respondent lived. A number of studies have found conflicting associations for hip fracture risk on the basis of geographic characteristics, including rurality (9-13). Northwest Texas has a few widely dispersed, large population centers surrounded by sparsely populated ranching and agricultural landscapes. No city in the region is truly urban compared with large metropolitan areas such as Dallas, San Antonio, and Houston. Some features, however, including convenient access to medical care, community hardscapes (e.g., walking trails, parks), commercial and municipal exercise facilities, and the availability of age-restricted housing, may be sufficiently distinct between the larger and smaller Northwest Texas counties to create a difference in the risk of hip fracture between these populations. For this study, we defined counties having a population greater than 100,000 as urban and counties with populations less than 100,000 as rural.

### Analysis

We used chi-square tests to compare case patients with control patients for the independent variables of interest. We then tested these explanatory variables for colinearity before including them in the final model. We used univariate and multivariate logistic regressions to test the associations between hip fracture and the independent variables of interest. The risk of hip fracture was estimated as an odds ratio (OR) with 95% confidence intervals (CI). We restricted analysis to respondents having complete data for the variables of interest.

To assess the overall fit of the logistic regression model, we examined differences between observed and expected frequencies of fracture in the study population by the Hosmer-Lemeshow goodness-of-fit statistic. The model was fitted on deciles of fracture risk (8) and was well calibrated and fit the data (Χ^2^_8_ = 11.05, *P* = .20). The overall fit of the model was confirmed by the receiver operating characteristic curve statistic of 0.857 with a standard error of 0.019. We used Stata 9.2 (StataCorp LP, College Station, Texas) for analyses and considered *P* < .05 statistically significant.

## Results

Of the study population of 190 case and 298 control patients, 56 (11.5%) reported currently smoking. Of the nonsmokers, 151 (30.9% of the total study population) were former smokers and 281 (57.6% of the total study population) had never smoked. We found no geographic gradient for smoking behavior. The percentage of current smokers among rural participants was 11.2%, and that among urban participants was 11.4%. The percentages of former smokers were also similar (32.8% for rural vs 29.4% for urban residents), as were the percentages of never smokers (56.0% for rural vs. 59.1% for urban residents).

Case patients differed significantly from control patients for most of the explanatory variables (Table 1). The mean age was 77.9 years (range, 50–101) for case patients and 63.7 years (range, 50–90) for control patients. Case patients were more likely than control patients to be current smokers (14.7% vs 9.4%) and to weigh less than 127 pounds (37.6% vs 16.6%). Only 25.3% of case patients reported exercising three or more times per week, whereas more than half of control patients (53.1%) did so. Case patients were less likely than control patients to have ever taken hormone replacement therapy (45.4% vs 68.0%), to be taking supplemental calcium (62.4% vs 75.7%), and to consume alcohol (17.6% vs 36.4%). Case patients were more likely than control patients to reside in rural settings (37.9% vs 18.6%).

In the multiple logistic regression model, current smoking was strongly associated with the risk of hip fracture, after adjustment for age and other potentially confounding variables (adjusted OR, 3.72; 95% CI, 1.59–8.70) (Table 2). Former smokers had an intermediate risk (adjusted OR, 2.27; 95% CI, 1.22–4.21), when compared with never smokers. As expected, insufficient exercise was a significant predictor of hip fracture in the adjusted model. Women who reported exercising fewer than three times per week were almost twice as likely as more active women to sustain a hip fracture (adjusted OR, 1.81; 95% CI, 1.04–3.15). Weight, age at menopause, use of hormone replacement therapy, and use of calcium supplements were not significantly associated with hip fracture.

Women living in a rural county were more likely than women living in an urban county to experience osteoporotic hip fracture (adjusted OR, 2.71; 95% CI, 1.48–4.95). We found no difference between the two populations in exercise levels: 42.8% of urban respondents and 39.7% of rural respondents reported exercising more than twice per week (*P* = .55). The percentage of women aged 80 years or older was higher in the rural (30.2%) than in the urban (20.1%) population (*P = *.01). Our findings also suggest that although these rural and urban populations had equal access to primary medical care (as measured by having a "regular family doctor" and by frequency of visits to a physician), women in rural counties were less likely to receive osteoporosis-related services, specifically BMD screening (56.2% vs 70.6%; *P* = .004). Women in rural counties were also less likely to take supplemental calcium (62.3% vs 73.9%; *P* = .02).

## Discussion

Geographic gradients in hip fracture risk reflect a complex interaction of the sociologic and physiologic disparities between urban and rural populations. Our finding that rural postmenopausal women were more likely than their urban counterparts to experience osteoporotic hip fracture is contrary to expectations based on our review of the literature.

Studies in Australia (9), Sweden (10), and Thailand (11), which found a higher rate of hip fracture in urban than in rural populations, hypothesized that higher levels of physical activity in rural populations lead to higher baseline BMD scores and, therefore, protect against hip fracture. One of the few U.S. studies of rural–urban gradients in hip fractures, conducted in Olmsted County, Minnesota, from 1980 through 1989 (12), found a 36% greater age- and sex-adjusted incidence of proximal femoral fractures among the residents of urban Rochester than among residents of rural Olmsted County. Higher average impact trauma for urban women was the suggested mechanism for this difference. A follow-up study examining data from the same populations for 1989 through 1991 (13) found that although the age- and sex-adjusted incidence of all fractures combined was 15% higher in the urban than in the rural population, the hip fracture gradient was only 6% and was not statistically significant.

Migration for care may have introduced some selection bias into our study and limited our ability to measure the effect of rurality on osteoporotic hip fracture. Although most residents of the rural counties in Northwest Texas are transferred to the regional medical center in Amarillo for surgical repair and rehabilitation of hip fracture, some choose treatment at a community hospital closer to home. Also, a higher percentage of control than of case patients might choose a primary care provider in or near their county of residence. Rural residents with complicated medical problems and serious comorbidity most likely would travel to a regional medical center for specialty care. This issue represents a layer of complexity that requires further research.

Numerous studies associate cigarette smoking with osteoporotic hip fracture. One large cohort study that followed more than 100,000 women for up to 12 years found that women who smoked 25 or more cigarettes per day had a relative risk of hip fracture 1.6 times higher than that of nonsmokers (95% CI, 1.1–2.3) (14). The risk for former smokers was lower, but the benefit was not observed until 10 years after cessation. Body weight confounded the effect of smoking. A Swedish case-control study also found that current smokers were at increased risk of hip fracture (age-adjusted OR, 1.66; 95% CI, 1.41–1.95) (15) and that this effect was magnified for postmenopausal smokers. In part, these findings reflect reduced BMD in smokers (6). BMD was similar for premenopausal smokers and nonsmokers in the Swedish study, but for each 10-year increment of age after menopause, the difference in BMD between smokers and nonsmokers increased by 2%. By age 80, the difference in BMD between the two groups was 6%, after adjusting for body size and exercise status. However, lower BMD accounts for only 23% of the smoking-related risk of hip fracture (8), and researchers have suggested other mechanisms to explain the remainder of the risk, including lower physical activity levels, higher comorbidity, earlier menopause, and changes to the microarchitecture of bone tissue in smokers (14-18).

Current statistics indicate that 8.4% of U.S. women older than 65 are active smokers (19). In Northwest Texas, 17.2% of women report current smoking, and 30% report smoking more than 100 cigarettes in their lifetime (20). Smoking is an addiction that typically begins during the teenage years (21), when a person's bones are forming. Our data show that unlike risk for cardiovascular disease due to smoking, which tends to return to baseline levels within several years after cessation (22), the risk for osteoporotic hip fracture remains significantly increased, even though relative risk is lower for former smokers than for smokers (2.27 vs 3.72).

Many risk factors for osteoporotic hip fracture, such as age, heredity, and body geometry, are not modifiable, but cigarette smoking is. Currently, physician education of patients who already have or who may develop osteoporosis usually includes recommendations for calcium and vitamin D supplementation and exercise. Smoking cessation should be equally emphasized in counseling both young and aging women, as part of an integrated approach to osteoporosis prevention. Consistent cessation messages from health care providers targeting patients who use tobacco are one of several evidence-based tobacco interventions recommended by the Task Force on Community Preventive Services (23). Unfortunately, only an estimated 50% to 60% of smokers receive this counseling (24). Our findings underscore the need for physician education of female patients who smoke — and especially of those who continue to smoke after menopause — as an important adjunct to osteoporosis screening and treatment in the prevention of osteoporotic hip fractures.

Although diagnosis of osteopenia and osteoporosis and subsequent pharmacotherapy may significantly benefit the high-risk individual, it will do little at the population level to stem the rising number of osteoporotic hip fractures. Population-based strategies targeting lifestyle changes may have less impact at the individual level, but they are more beneficial to the overall health outcomes of large populations (25). In effect, the goal of health intervention should be to shift the mean of the whole population in the direction of better health. In the case of osteoporotic hip fracture, achieving this goal requires a reduction of modifiable osteoporotic risk factors. Smoking, one of these modifiable risk factors, appears to result in a significant risk gradient in our study population of postmenopausal women residing in Northwest Texas.

## Figures and Tables

**Table 1:** Smoking and Other Characteristics of Case and Control Patients (N = 488)^a^, Hip Fracture Study, Northwest Texas, July 2004–April 2006

Characteristic	Sample n (%)	Case Patients, % (n = 190)	Control Patients, % (n = 298)	*P* value
**Smoking status**				
Never smoked	281 (57.6)	52.1	61.1	.08
Former smoker	151 (30.9)	33.2	29.5	
Current smoker	56 (11.5)	14.7	9.4	
**Age, y**				
50-64	187 (39.0)	11.6	56.7	<.001
64-79	183 (38.1)	43.4	34.7	
≥80	110 (22.9)	45.0	8.6	
**Weight, lb**				
<127	116 (25.0)	37.6	16.6	<.001
≥127	347 (75.0)	62.4	83.4	
**No. times exercise/wk**				
<3	274 (57.8)	74.7	46.9	<.001
≥3	200 (42.2)	25.3	53.1	
**Age at menopause, y**				
≤34	55 (13.2)	13.9	12.8	.36
35-44	117 (28.1)	30.3	26.7	
45-51	166 (39.9)	41.2	39.0	
≥52	78 (18.8)	14.6	21.5	
**Hormone replacement**				
Ever	277 (59.1)	45.4	68.0	<.001
Never	192 (40.9)	54.6	32.0	
**Calcium supplementation**				
Yes	331 (70.4)	62.4	75.7	.002
No	139 (29.6)	37.6	24.3	
**Rurality**				
Urban	350 (73.7)	62.1	81.4	<.001
Rural	125 (26.3)	37.9	18.6	
**Alcohol consumption**				
Yes	139 (29.0)	17.6	36.4	<.001
No	340 (71.0)	82.4	63.6	

a Some categories do not add to 488 because of missing data.

**Table 2:** Determinants of Hip Fracture Risk, Univariate and Multivariate Logistic Regression, Hip Fracture Study (N = 366)^a^, Northwest Texas, July 2004–April 2006

Characteristic	Unadjusted OR (95% CI)	Multivariate OR (95% CI)
**Smoking status**		
Never smoked	1.00	1.00
Former smoker	1.32 (0.88-1.97)	2.27 (1.22-4.21)
Current smoker	1.84 (1.03-3.28)	3.72 (1.59-8.70)
**Age, y**		
50-64	1.00	1.00
64-79	5.90 (3.48-10.01)	6.37 (3.29-12.34)
≥80	25.11 (13.45-46.89)	25.88 (10.55-63.46)
**Weight, lb**		
<127	3.07 (2.00-4.72)	1.10 (0.58-2.09)
≥127	1.00	1.00
**No. times exercise/wk**		
<3	3.33 (2.22-4.98)	1.81 (1.04-3.15)
≥3	1.00	1.00
**Age at menopause, y**		
≤34	1.00	1.00
35-44	1.04 (0.54-1.99)	0.89 (0.37-2.14)
45-51	0.96 (0.51-1.77)	0.81 (0.35-1.87)
≥52	0.62 (0.30-1.27)	0.46 (0.18-1.22)
**Hormone replacement**		
Ever	1.00	1.00
Never	2.56 (1.75-3.76)	1.74 (0.99-3.06)
**Calcium supplementation**		
Yes	1.00	1.00
No	1.88 (1.26-2.80	1.53 (0.82-2.83)
**Rurality**		
Urban	1.00	1.00
Rural	2.53 (1.67-3.82)	2.71 (1.48-4.95)
**Alcohol consumption**		
Yes	1.00	1.00
No	2.69 (1.72-4.20)	1.63 (0.86-3.06)

OR indicates odds ratio; CI, confidence interval.

a Analysis restricted to respondents who had complete data for variables of interest.

## References

[1] (2004). U.S. interim projections by age, sex, race, and Hispanic origin.

[2] McBean LD, Forgac T,  Finn SC (1994). Osteoporosis visions for care and prevention — a conference report. J Am Diet Assoc.

[3] Marshall D, Johnell O, Wedel H (1993). Meta-analysis of how well measures of bone mineral density predict occurrence of osteoporotic fractures. BMJ.

[4] Law MR, Hackshaw AK (1997). A meta-analysis of cigarette smoking, bone mineral density and risk of hip fracture: a recognition of a major effect. BMJ.

[5] Gregg EW, Pereira MA, Caspersen CJ (2000). Physical activity, falls, and fractures among older adults: a review of the epidemiologic evidence. J Am Geriatr Soc.

[6] Egger P, Duggleby S, Hobbs R, Fall C, Cooper C (1996). Cigarette smoking and bone mineral density in the elderly. J Epidemiol Community Health.

[7] Bjarnason NH, Christiansen C (2000). The influence of thinness and smoking on bone loss and response to hormone replacement therapy in early postmenopausal women. J Clin Endocrinol Metab.

[8] Kanis JA, Johnell O, Oden A, Johansson H, De Laet C, Eisman JA (2005). Smoking and fracture risk: a meta-analysis. Osteoporos Int.

[9] Sanders KM, Nicholson GC, Ugoni AM, Seeman E, Pasco JA, Kofowicz MA (2002). Fracture rates lower in rural than urban communities: the Geelong Osteoporosis Study. J Epidemiol Community Health.

[10] Ringsberg KA, Gardsell P, Johnell O, Jonsson B, Obrant KJ, Sernbo I (1998). Balance and gait performance in an urban and a rural population. J Am Geriatr Soc.

[11] Pongchaiyakul C, Nguyen TV, Kosulwat V, Rojroongwasinkul N, Charoenkiatkul S, Rajatanavin R (2005). Effect of urbanization on bone mineral density: a Thai epidemiological study. BMC Musculoskelet Disord.

[12] Madhok R, Melton LJ, Atkinson EJ, O'Fallon WM, Lewallen DG (1993). Urban vs rural increase in hip fracture incidence. Age and sex of 901 cases 1980-89 in Olmsted County, U.S.A.. Acta Orthop Scand.

[13] Melton LJ, Crowson CS, O'Fallon WM (1999). Fracture incidence in Olmsted County, Minnesota: comparison of urban with rural rates and changes in urban rates over time. Osteoporos Int.

[14] Cornuz J, Feskanich D, Willett WC, Colditz GA (1999). Smoking, smoking cessation, and risk of hip fracture in women. Am J Med.

[15] Baron JA, Farahmand BY, Weiderpass E, Michaelsson K, Alberts A, Persson I (2001). Cigarette smoking, alcohol consumption and risk of hip fracture in women. Arch Intern Med.

[16] Gregg EW, Pereira MA, Caspersen CJ (2000). Physical activity, falls, and fractures among older adults: a review of the epidemiologic evidence. J Am Geriatr Soc.

[17] Hoidrup S, Sorensen TI, Stroger U, Lauritzen JB, Schroll M, Gronbaek M (2001). Leisure-time physical activity levels and changes in relation to risk of hip fracture in men and women. Am J Epidemiol.

[18] Farahmand BY, Persson PG, Michaelsson K, Baron JA, Alberts A, Moradi T (2000). Physical activity and hip fracture: a population-based case-control study. Swedish Hip Fracture Study Group. Int J Epidemiol.

[19] Smoking among adults: U.S. 2000–2005. In: Health data for all ages.

[20] Behavioral Risk Factor Surveillance System, data table lookup (data for 2005, Public Health Administrative Region 1).

[21] Johnson LD, O'Malley PM, Bachman JG, Schulenberg JE (2006). Monitoring the future: national survey results on drug use 1975–2005. Vol. 1: secondary school students.

[22] (2000). Reducing tobacco use: a report of the Surgeon General.

[23] (2005). Effectiveness of using provider reminders and provider education with or without patient education. In: Tobacco, guide to community preventive services.

[24] Hopkins DP, Briss PA, Ricard CJ, Husten CG, Carande-Kulis VG, Fielding JE (2001). Reviews of evidence regarding interventions to reduce tobacco use and exposure to environmental tobacco smoke. Am J Prev Med.

[25] Rose G (1985). Sick individuals and sick populations. Int J Epidemiol.

